# Pancreaticogastrostomy as reconstruction choice in pancreatic trauma surgery: Case report and review of the literature

**DOI:** 10.1016/j.ijscr.2019.10.030

**Published:** 2019-10-22

**Authors:** Francesco Serra, Giuseppe Barbato, Giovanni Tazzioli, Roberta Gelmini

**Affiliations:** Department of Surgery, University of Modena and Reggio Emilia – Policlinico of Modena, Via del Pozzo, 71, 41100 Modena, Italy

**Keywords:** Pancreatic trauma management, Parenchymal sparing pancreatic surgery

## Abstract

**Introduction:**

Due to its retroperitoneal location and its proximity to major vascular structures and other organs, isolated pancreatic injuries are rare. The optimal management for pancreatic injuries of grades III and IV, where a main ductal transection is present, remains controversial. Isolated complete traumatic transection of the pancreatic neck is uncommon, but this condition is associated with some peculiar technical aspects that allow more conservative treatments.

**Presentation of the case:**

A closed abdominal blunt trauma in a young patient underwent emergency surgery for suspect hemoperitoneum. Intraoperatively evidence of complete traumatic transection of the pancreatic neck treated with pancreas tissue debridement, suture of the cephalic stump and pancreaticogastrostomy reconstruction.

**Discussion:**

Preservation of pancreatic volume and avoidance of adjacent organ resection is associated with lesser mortality and morbidity rate. The advantages of conservative treatments are related to reductions in the postoperative exocrine and endocrine insufficiencies.

**Conclusion:**

In selected cases of complete neck transection with preserved pancreatic parenchyma in a stable patient, parenchymal-sparing interventions should be considered. Pancreaticogastrostomy offers an easier to learn and faster technique also suited for less experienced surgeons.

## Highlights

• Pancreatic trauma with complete wirsung transection in man treated with pancreaticogastrostomy reconstruction in emergency.
• High risk of complications like postoperative pancreatic fistula (POPF).
• The need of protocols for correct manage of this patients in emergency surgery shared by surgeons.

## 1 Introduction

Traumatic injuries to the pancreas are uncommon but can be associated with major morbidity and mortality including acute haemorrhage, pancreatic leaks, abscesses, fistulae, and pancreatitis [2]. Estimates for the incidence of pancreatic injury range from 0.2% to 12% of abdominal traumas [[3], [4], [5], [6], [7]].

Motor vehicle collisions cause approximately 75–85% of blunt injuries to the duodenum and pancreas. The mechanism is typically due to the crushing of these fixed retroperitoneal organs between the vertebral column and steering wheel [8].

Due to its retroperitoneal location and its proximity to major vascular structures and other organs, isolated pancreatic injuries are rare. The reported numbers for isolated pancreatic injuries have been as low as 0.7% for blunt injury [9]. The American Association for the Surgery of Trauma (AAST) grading system, published in 1990, is a practical and prognostic way to describe the pancreatic injury, higher-grade injuries correlate with higher mortality and complications. A most important factor affecting outcome after a pancreatic injury is the presence or absence of main pancreatic duct injury. AAST classification for pancreatic injury grades the severity of injury based on the location of the lesion in the pancreas (proximal versus distal) and the presence of main pancreatic duct injury [10]. There are several approaches for dealing with the injured pancreas, ranging from drainage only, suture repair, or pancreatic resection with or without immediate reconstruction. The optimal management for pancreatic injuries of grades III and IV, where a main ductal transection is present, remains controversial. The Eastern Association for Surgery of Trauma conditionally recommends distal pancreatectomy with or without splenectomy for adult patients who are undergoing surgery [11]. Isolated complete traumatic transection of the pancreatic neck is rare but is associated with some peculiar technical aspects that allow more conservative treatments than distal pancreatectomy [12].

## 2 Presentation of the case

A 22-year-old man comes to our observation after having reported a non-commotional facial skull injury with multiple facial lacerated wounds and a closed abdominal trauma caused by a vehicle crash with a steering wheel impact. The patient is alert and oriented; the vital parameters are stable, reports abdominal pain to the upper quadrants. Laboratory tests showed leukocytosis (23,000/mm^3^) hyperamylasemia (743 U/L), hypertransaminasemia (sGOT 547, sGPT 443), no signs of anaemia. An ultrasound examination for the evaluation of injured patients (Focused Assessment with Sonography for Trauma - FAST) shows an inhomogeneous image of the inferior spleen pole and a modest free fluid suspected for hemoperitoneum. Evaluated the dynamic of trauma, that was a major trauma caused from an impact with high speed, the FAST that shown fluid suspected for hemoperitoneum and the spleen with inhomogeneous lower pole, the decision was to proceed directly to the operating room. Therefore, the patient underwent a surgical emergency procedure that points out the complete transection of the pancreatic neck without mesenteric vessels or duodenum lesions, absence of spleen trauma or other associated injuries. The proximal pancreatic stump was sutured with a separated ligation of the Wirsung duct. The Wirsung duct was not recognisable at the level of the distal pancreatic stump; this is the reason why a pancreaticogastrostomy between the distal pancreatic segment and the posterior wall of the stomach was performed, associated with a decompressive gastrostomy. A nutritional jejunostomy was performed too (Table 1).

**Table 1:** Pros and cons of PG and PJ construction.

	PROS	CONS
PG RECONSTRUCTION	Favourable topographic anatomy	High risk of postoperative bleeding
	Speed of handling in emergency setting	No clear evidence of reduce POPF
	Abundant stomach wall vascularisation	
	Low risk of anastomosis dehiscence	
	Easier to learn for young surgeons in emergency settings	

PJ RECONSTRUCTION	Low risk of post-operative bleeding	High risk of intrabdominal abscess
	Most common reconstruction	No clear evidence of reduce POPF

**Fig. 1:**
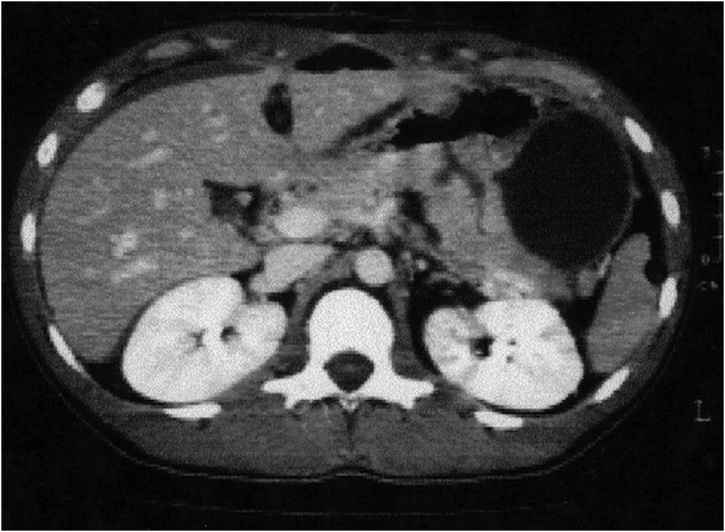
The CT-SCAN show the PG reconstruction and the pseudocyst that is treated with a percutaneous radiological drainage.

The postoperative course was complicated by the appearance of melena on postoperative day (POD) 6th for which the patient underwent an EGDS that show self-limiting bleeding at the pancreaticogastrostomy (PG) managed conservatively. On POD 10th, for the appearance of fever and bowel obstruction, the patient performed a Computed tomography (CT) scan that highlighted a 12 cm pseudocyst in the left upper quadrant between stomach and spleen. The developed pseudocyst was managed conservatively with a CT guided drainage (Fig. 1).

The subsequent course did not show further complications; to date, the patient presents a complete functional recovery. Two CT scans were performed after one month and four months after surgery showed the complete resolution of the abdominal injury without sequelae. It should be reported a reduction of the pancreatic tail dimensions while the head appears normal in a context of the clinical absence of exocrine or endocrine gland insufficiency. A ten year follow up visit the patient is in good health, and no metabolic disorders have acquired.

## 3 Discussion

Higher-grade injuries involving the pancreatic duct have increased morbidity and mortality as well as the potential for deterioration if treatment is delayed, and literature supports resection in these cases [13]. CT scan is the diagnostic modality of choice in hemodynamically stable blunt abdominal trauma patients to diagnose the pancreatic injury. The TC-scan sensitivity for detecting pancreatic injury is highly variable ranging from 47% to 79%. Identification of pancreatic duct injury using CT imaging also varied, with sensitivities ranging from 52% to 54% with specificities between 90% and 95%. The use of magnetic resonance cholangiopancreatography (MRCP) imaging is believed to increase the diagnostic confidence of pancreatic duct injury [14]. MRCP can be a useful tool for diagnostic purposes, whereas endoscopic retrograde cholangiopancreatography (ERCP) may provide diagnostic as well as a therapeutic intervention but is limited due to the logistics of performing ERCP in general and the technical challenges of performing it in a multiple trauma patient with the risk of exacerbating the issue with pancreatitis [15]. In any cases MRCP is not considered fundamental in emergency care. Our operative management with the parenchymal sparing technique was based on the pancreatic injury grade also considering the absence of other associated injuries, the clinical conditions and the physiological status of the patient. We hypothesize that non-resection management is associated with lesser mortality and morbidity rate, and shorter hospital length of stay. Limiting the number of unnecessary distal resections performed thus limiting the number of inherent complications. Other advantages of conservative treatments are related to reductions in the postoperative exocrine and endocrine insufficiencies.

Pancreas tissue debridement, suturing the cephalic stump and PG reconstruction given preservation of pancreatic volume and avoidance of adjacent organ resection. The most common reconstruction technique among the cases reported in the literature is the suture of the cephalic remnant and the reconstruction of the left remnant with a Roux-en-y pancreatic-jejunal anastomosis; however, it is possible to perform a PG and also the direct duct-to-duct reconstruction over a stent [16].

In our case, we chose to perform a PG reconstruction. The best method to restore pancreatic digestive continuity is still debated. End to side PJ is the most common reconstruction after pancreaticoduodenectomy but, as compared to PJ, the anastomosis between the pancreatic remnant and the stomach provides several putative advantages as shown in table.

**Fig. 2:**
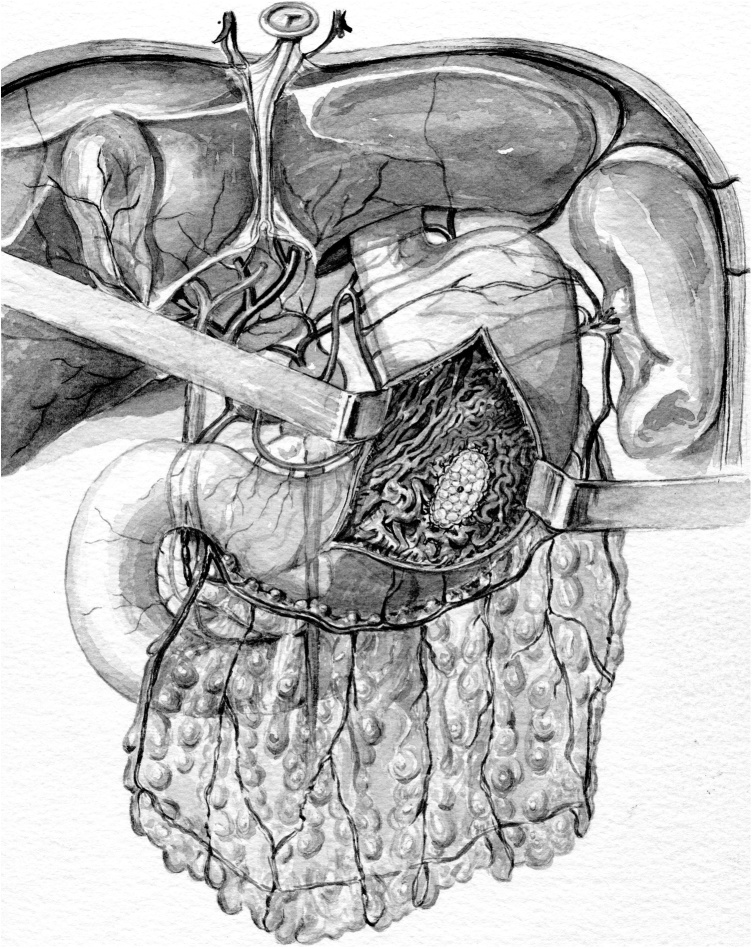
The picture show the PG reconstruction.

**Fig. 3:**
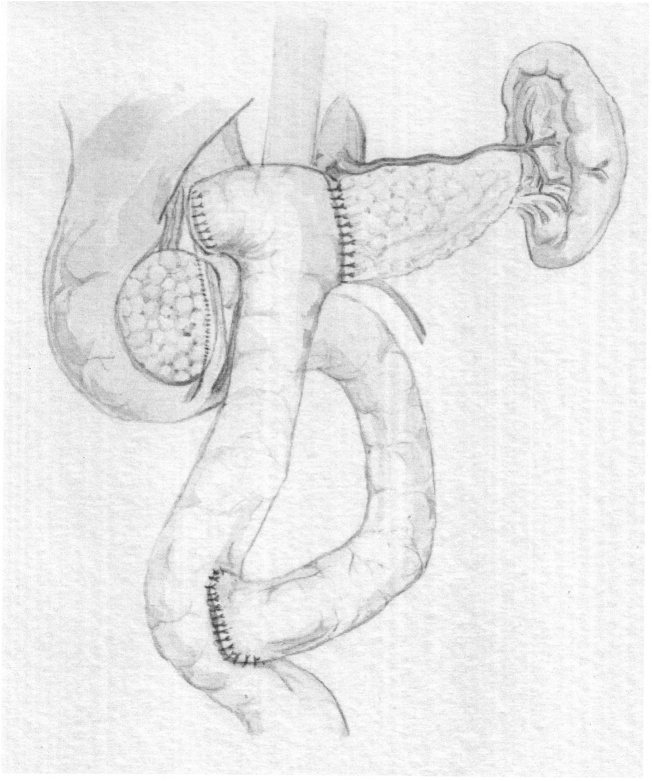
Schematic picture of PJ reconstruction after the trauma of the pancreatic neck.

However, the parenchymal sparing techniques seem to determine a lower mortality rate and a lower incidence of re-operation [11,17,18]. Pancreaticoduodenectomy still has high overall morbidity, principally due to a clinically relevant postoperative pancreatic fistula (POPF) [19]. Despite numerous reports and trials describing novel methods to curtail the risk of POPF formation, the reported rates of POPF have not significantly improved over the last years (Fig. 2, Fig. 3).

There are several Randomized controlled trials (RCT) and Meta-analysis that have been published on this topic, results are often discordant and clear evidence on the ideal management, and surgical technique to reduce POPF rate is not yet provided. Many confounding factors were considered to influence the development of postoperative pancreatic fistula (e.g. age, obesity, cardiovascular diseases, diabetes mellitus, pancreatic texture, pancreatic duct size). Other risk factors that potentially may affect POPF, such as pancreatic duct stenting, octreotide, different types of procedures and extent of resection, were not distributed homogeneously among the studies [20].

A 2017 Cochrane review compare PG versus PJ to prevent the POPF after pancreaticoduodenectomy [21]. There was little or no difference between PJ and PG in the overall risk of POPF (grades A, B or C) and clinically significant POPF (grades B or C). Risk of postoperative bleeding was lower in participants undergoing PJ, but this benefit was offset by a higher risk of developing an intra-abdominal abscess associated with the PJ procedure.

PG, it may be technically easier for novice surgeons to construct a secure, especially with a soft pancreas [22].

Fernandez trial was the first to report a significantly lower incidence of CR-POPF with a PG (4% in PG and 18% in PJ, P < .001). It is important to emphasize that they used a gastric partition technique with preservation of the gastroepiploic arcade which is technically complex and not always possible oncologically [23].

Topal study used a stratified design based on the pancreatic ductal diameter (<3 mm vs >3 mm) and showed a significantly lower rate of CR-POPF in the PG group (odds ratio [OR] 2.86, 95% confidence interval [CI] 1.38–6.17; P = .002). In patients with duct diameter of less than or equal to 3 mm, the CR-POPF rate was 24.5% in the PJ group vs 10.2% in the PG group; however, this apparent improvement did not translate into a statistically significant decrease in morbidity for unclear reasons [24].

Figueras et al. reported a significantly lower incidence of CR-POPF in the PG group when compared to the PJ group (11% vs 33%, P = .006) [25].

The limited number of the cases in emergency context makes more difficult to establish the best surgical strategy; however, recently, some authors have suggested an algorithm for the management of pancreatic trauma that could help the surgeons during the decision-making process [26].

## 4 Conclusion

Although the pancreas remains one of the least frequently injured organs in cases of blunt abdominal trauma, its location and the fact that vital structures surround it means that pancreatic injuries often present complicated diagnostic and treatment problems. When a complete Wirsung duct transection is present, treatment requires surgical intervention in the majority of cases. Distal pancreatectomy with or without splenectomy is the treatment of choice, but in cases of complete neck transection with preserved pancreatic parenchyma, parenchymal-sparing interventions should be considered in a stable patient. Our surgical management focused on minimising not only the short-term morbidity and mortality but also the long-term functional outcome. PG offers an easier to learn and faster technique also suited for less experienced surgeons, but there is no reliable evidence to support the use of PG over PJ. However, limitations such as inadequate power, lack of direct comparison between groups, incomplete reporting of mortality and bias in literature reflect the fact that the technique used for management of the pancreatic injury is still a matter of debate.

The work was written in line with the SCARE criteria [1].

## Funding

Non funding were used.

## Ethical approval

This study is exempt from ethical approval in our institution.

## Consent

An informed written consent to use data for clinical research and publication was given at the hospital admission.

## Author’s contribution

Serra Francesco, MD: Data collection and author of case report and discussion.

Barbato Giuseppe, MD: Review of surgical technique literature and co-author.

Tazzioli Giovanni, MD: Co-author of discussion.

Gelmini Roberta, MD PhD: Supervisor and co-author of entire manuscript.

## Registration of Research Studies

N/A.

## Guarantor

Gelmini Roberta.

## Provenance and peer review

Not commissioned, externally peer-reviewed.

## Declaration of Competing Interest

No conflicts of interest.
